# Association between household exposure and cycle threshold in COVID-19 infected health care workers

**DOI:** 10.1186/s12995-021-00321-3

**Published:** 2021-08-04

**Authors:** Ai Chien, Sandra Domeracki, Sandeep Guntur, Kristopher Taylor, Chuanyi M. Lu, Harry Lampiris, Paul D. Blanc

**Affiliations:** 1 Occupational and Employee Health Section and Infectious Disease Section, Medical Service and Laboratory Medicine Service, San Francisco Veterans Affairs Health Care System, 4150 Clement St., San Francisco, CA 94121 USA; 2 Division of Occupational and Environmental Medicine, Department of Medicine, University of California San Francisco, San Francisco, USA; 3 Occupational and Environmental Health Nursing, Community Health Systems, School of Nursing, University of California San Francisco California, San Francisco, USA; 4 Department of Laboratory Medicine, University of California San Francisco, San Francisco, USA; 5 Division of Infectious Disease, Department of Medicine, University of California San Francisco, San Francisco, USA

**Keywords:** COVID-19, Health care workers, Household exposure, Cycle threshold

## Abstract

**Objective:**

Household SARS-COV-2 contact constitutes a high-risk exposure for health care workers (HCWs). Cycle threshold (Ct) of reverse transcriptase–polymerase chain reaction testing provides an estimate of COVID-19 viral load, which can inform clinical and workplace management. We assessed whether Ct values differed between HCWs with COVID-19 with and without household exposure.

**Methods:**

We analyzed HCW COVID-19 cases whose Ct data could be compared. We defined low Ct at a cut-point approximating a viral load of 4.6 × 10^6^ copies per ml. Logistic regression tested the association of household exposure and symptoms at diagnosis with a low Ct value.

**Results:**

Of 77 HCWs with COVID-19, 20 were household exposures cases and 34 were symptomatic at testing (7 were both household-exposed and symptomatic at testing). Among household exposures, 9 of 20 (45%) manifested lower Ct values compared to 14 of 57 (25%) for all others. In a bivariate model, household exposure was not statistically associated with lower Ct (Odds Ratio [OR] 1.20; 95% Confidence Interval [CI] 0.97–1.51). In multivariable modelling both household exposure (OR] 1.3; 95% CI 1.03–1.6) and symptoms at diagnosis (OR 1.4; 95% CI 1.15–1.7) were associated with a low Ct value.

**Discussion:**

Household exposure in HCWs with newly diagnosed COVID-19 was associated with lower Ct values, consistent with a higher viral load, supporting the hypothesis that contracting COVID-19 in that manner leads to a greater viral inoculum.

## Introduction

Cycle threshold (Ct) of reverse transcriptase–polymerase chain reaction, the number of amplification cycles for the target gene to exceed threshold detection, can provide an estimate of SARS-COV-2 viral load and may be related to infectivity and disease severity [1]. Nonetheless, Ct quantification can be fraught with variability, especially across laboratories, and is not currently recommended as a measure to inform clinical management [2]. Among health care workers (HCWs) with COVID-19, Ct data have been analyzed in light of return-to-work protocols in one study and used in analysis of work-absence risk in another [3, 4].

Household exposure to SARS-COV-2 has been associated with a higher secondary attack rate than other sources of exposure, including acquisition in health care settings [5, 6]. This presumably reflects a greater intensity of exposure to the virus in persons acquiring COVID-19. In a cohort of HCWs with acute SARS-COV-2COVID-19 infection and with well-characterized contact tracing, we assessed whether the Ct differed between those with illness likely contracted from a household member compared to all others.

## Methods

As part of clinical quality improvement activities at the San Francisco Veterans Affairs Healthcare System, we analyzed positive SARS-COV-2 RT-PCR testing (nasopharyngeal or oropharyngeal swab). These included both symptomatic and asymptomatic employees (the latter tested due to exposure or in routine surveillance monitoring). We limited analysis to cases March 2020 through January 2021 whose initial positive testing was performed at the San Francisco Veterans Affairs Healthcare System laboratory using the same Abbott RealTime SARS-COV-2 assay providing directly comparable Ct values. The Abbott assay is United States Food and Drug Administration Emergency Use Authorization-approved as a qualitative test and was calibrated in-house using standard reference materials [3]. We excluded cases initially diagnosed with COVID-19 by use of other assays at our site or elsewhere.

We reviewed our employee health unit’s COVID-19 database, using narrative information to identify whether infection was attributable to residing with someone whose COVID-19 preceded that of the employee. The database narrative text, in all cases, had been entered by one of four trained clinicians (two physicians and two advanced practice nurses) at the time of intake by interviewing the cases and was supplemented as appropriate by a nurse who carried out contact tracing. The interview specifically focused on potential sources of exposure, including any household contacts who were symptomatic or diagnosed with SARS-COV-2. The designation of a household source required that the narrative indicated onset of symptoms or diagnosis preceding the onset in the employee case in a time period consistent with the COVID-19 incubation period (2 to 14 days). A single researcher (author AC) categorized the employees on this basis, with adjudication when questions arose by another (author SD) using supplemental information garnered through the return-to-work process for ill employees.

We also determined if the employee was symptomatic at initial testing. We defined symptomatic as log documentation of the employee reporting any of a standard check list of COVID-19 related complaints we routinely used to categorize any employees as a “person under investigation” (PUI). These included: a body temperature of 100 degrees Fahrenheit or greater, malaise or muscle ache, upper or lower respiratory complaints, loss of smell or taste, other neurological symptoms, gastrointestinal upset, or dermal or ocular complaints.

Because we anticipated that symptomatic status at the time of initial test positivity might be associated with a higher estimated viral load, we wanted to assess any confounding association between such symptomatic status and a household source of exposure. To do so, we tested the cross-tabulation of symptomatic status vs. household exposure (both defined dichotomously) using the Chi square. We defined low Ct (indicative of higher viral load) as a value below the lowest quartile cutoff among the non-household contact cases: a Ct of 9.32 (estimated viral load approximating 4.6 × 10^6^ copies per ml). We chose the lowest quartile rather than the median value of the referent HCWs (those without a household source) to reflect the lower threshold “tail” of these non-normally distributed data. Our laboratory standard curve established a linear correlation of lower Ct to higher viral load with this lower quartile cut-off being multiple orders of magnitude above the reliable limit of detection (the positive/negative test report used clinically). Logistic regression tested the association of household exposure alone or adjusted for symptomatic case status with low Ct (RStudio 2021; PBC, Boston, MA).

## Results

Of 141 SARS-COV-2 infected employees, 64 were excluded because they did not have initial diagnostic testing performed with the Abbott RealTime at the SFVAHCS laboratory. Of 77 analyzed, 20 (26%) were household exposures. Of these, 7 of 20 (35%) were symptomatic at the time of initial testing, whereas 13 of 20 (65%) were asymptomatic. Another 26 were symptomatic but did not have a household exposure source (total symptomatic, 33 of 77 [44%]). The association between having a household exposure source and being symptomatic at the time of testing was not statistically significant (*p* > 0.3). Of 20 household exposure cases, 9 (45%) manifested lower Ct values; whereas 14 of 57 (25%) non-household exposure cases had lower Ct values. Table 1 presents the results of two logistic regression models. A bivariate model presents household exposure as the sole risk factor for a lower Ct value of a positive SARS-COV-2 PCR test. In that model the association is not statistically significant. The second, multivariable analysis presents a model including both household exposure and being symptomatic at the time of testing, even though these two potential risk factors were not statistically associated with each other as previously presented. In the multivariable model, the association is marginally statistically significant.

**Table 1:** Odds of Low Cycle Threshold (Ct) Among 77 COVID-19 Positive Health Care Workers

Logistic Regression Model	Risk Factor (no. with risk factor)	Odds Ratio	95% CI
Model 1 Household Exposure Alone in Bivariate Model of Low Ct	Household Exposure (20)	1.2	0.97–1.5
Model 2 Multivariable Model of Low Ct including Household Exposure and Symptomatic at Test	Household Exposure (20)	1.3	1.03–1.6
	Symptomatic at Time of Test (34)	1.4	1.15–1.7

*No*. Number; *CI* Confidence Interval

## Discussion

Household exposure was associated with lower Ct, and therefore a higher viral load. This supports the hypothesis that contracting illness through household exposure represents a greater viral inoculum than other HCWs with acute SARS-COV-2 infection but without this exposure scenario. This could be related to multiple potential factors (prolonged frequency and duration of exposure, no mask wearing at home, lack of physical distancing, inadequate ventilation). Of course, we cannot exclude the possibility that some of the HCWs with household exposure might also have had a simultaneous, independent additional exposure to SARS-COV-2 either at the workplace or the community.

The association of household source to lower Ct was clarified taking into account being symptomatic, reflecting better model performance when considering both factors together when each was associated with increased odds of the same outcome. Being symptomatic has been shown to be related to lower Ct [7]. Limiting analysis to HCWs with COVID-19 has the advantage of studying a cohort with consistent access to testing and with thorough, standardized contact tracing to ascertain that the household was the likely source of exposure. Nonetheless, this does limit generalizability. A study of HCWs in Switzerland, although it did not analyze Ct data, reported higher risk of seropositivity associated with household but not healthcare-associated SARS-COV-2 contact [8]. Beyond HCWs, studies of professional sports cohorts have reported Ct data, but have not analyzed the relationship between estimated viral load and likely exposure source [ 9, 10]. We also acknowledge the limitation that HCWs with COVID-19 but without a household source of infection served as the referent in our analysis. There was no other appropriate comparison group from within our clinical laboratory as the patient population served was quite older and had underlying comorbidities. Another limitation of this study is its relatively small sample size, although this does not account for the statistical associations we observed. Nonetheless, any non-systematic misclassification of risk factors that would weaken associations (widening confidence intervals) would be magnified in a relatively small study cohort. The association of household exposure with lower SARS-COV-2 Ct that we observed suggests that greater viral inoculum could lead to a higher viral load in infection and may provide insight into this disease among HCWs. This could, if confirmed in other studies, inform case surveillance, contact tracing, and other aspects of occupational case management, with the caveat that given its variability, Ct should not guide clinical management.

## Data Availability

Data sharing is not applicable to this article as no datasets were generated or analyzed during the current study.

## Abbreviations

HCW: Health care work
Ct: Cycle threshold
OR: Odds ratio
CI: Confidence Interval
